# Draft Genome Sequence of *Photorhabdus luminescens* Strain BA1, an Entomopathogenic Bacterium Isolated from Nematodes Found in Egypt

**DOI:** 10.1128/genomeA.00396-14

**Published:** 2014-05-01

**Authors:** Shimaa Ghazal, Sheldon G. Hurst, Krystalynne Morris, Feseha Abebe-Akele, W. Kelley Thomas, Usama M. Badr, Mona A. Hussein, Mohamed A. AbouZaied, Kamal M. Khalil, Louis S. Tisa

**Affiliations:** a University of New Hampshire, Durham, New Hampshire, USA; b Applied Microbial Genetics Laboratory, Genetics and Cytology Department, Genetic Engineering & Biotechnology Division, National Research Centre, Dokki, Cairo, Egypt; c Department of Pests and Plant Protection, Agricultural Division, National Research Centre, Dokki, Cairo, Egypt; d Department of Microbiology, Faculty of Science, University of Ain Shams, Abbassia, Cairo, Egypt

## Abstract

*Photorhabdus luminescens* strain BA1 is an entomopathogenic bacterium that forms a symbiotic association with *Heterorhabditis* nematodes. We report here a 5.0-Mbp draft genome sequence for *P. luminscens* strain BA1, with a G+C content of 42.46% and 4,250 candidate protein-coding genes.

## GENOME ANNOUNCEMENT

Members of the genus *Photorhabdus* are Gram-negative motile bioluminescent bacteria that maintain two distinct lifestyles as insect pathogens and as symbionts with entomopathogenic *Heterorhabditis* nematodes (for reviews, see references 1–7). The life cycle of *Photorhabdus* and its nematode host *Heterorhabditis* is best described as a cyclic association that begins and ends with infective juvenile nematodes (IJs). The nonfeeding third-instar infective stage nematode retains a monoculture of *Photorhabdus* within the anterior region of the intestine (8, 9). The nematodes actively seek and infect insect hosts by entering through natural openings or by burrowing directly through the insect cuticle. Once inside the insect, the nematodes regurgitate the bacteria into the hemolymph (8). The bacteria release highly virulent toxins (10, 11), which results in insect death in <48 h. As the bacteria enter the stationary phase of their growth cycle, they secrete extracellular enzymes that aid in breaking down insect tissue, thereby providing nutrients for both the bacteria and nematodes. The bacteria also generate essential growth factors for nematode growth and development. The growth and development of *Heterorhabditis* nematodes have an obligate requirement for their specific bacterial symbiont (12). The bacteria also release antibiotics to prevent secondary invasion and putrefaction of the insect carcass (13, 14). After several days of feeding, the nematodes and bacteria reassociate and leave in search of a new insect host.

Based on molecular analysis, the *Photorhabdus* genus is divided into three bacterial species: *Photorhabdus luminescens*, *Photorhabdus temperata*, and *Photorhabdus asymbiotica* (15, 16). Our understanding of these bacteria has been greatly enhanced by the genome sequencing of two of the three established species: *P. luminescens* TT01 (17) and *P. asymbiotica* ATCC 43949 (18, 19). Recently, draft genomes have been available for *P. asymbiotica* Kingcliff (20) and *P. temperata* M121 (21). Here, we present a draft genome sequence for *P. luminescens* strain BA1, which was isolated from *Heterorhabditis bacteriophora* (BA1) nematodes found in Egypt (22, 23).

The draft genome of *P. luminescens* BA1 was generated at the Hubbard Genome Center (University of New Hampshire, Durham, NH) using Illumina technology (24) techniques. A standard Illumina shotgun library was constructed and sequenced using the Illumina HiSeq 2000 platform, which generated 41,799,700 reads (260-bp insert size) totaling 4,179.9 Mbp. The Illumina sequence data were assembled using the CLC Genomics Workbench (6.5.1) and AllPaths-LG (version r41043) (25). The final draft assembly contained 114 contigs, with an *N*_50_ of 60.9 kb. The total size of the genome is 5.0 Mbp, and the final assembly is based on 3,341 Mb of Illumina draft data, which provided an average 668.2× coverage of the genome.

The high-quality draft genome of *P. luminescens* strain BA1 was resolved to 114 contigs consisting of 5,004,588 bp, with a G+C content of 42.46%. The assembled *P. luminescens* BA1 genome was annotated via the Integrated Microbial Genomes (IMG) platform developed by the Joint Genome Institute, Walnut Creek, CA, USA (26), and resulted in 4,250 candidate protein-coding genes.

### Nucleotide sequence accession numbers.

This whole-genome shotgun project has been deposited at DDBJ/EMBL/GenBank under the accession no. JFGV00000000. The version described in this paper is version JFGV01000000.
